# Teens online: how perceived social support influences the use of the Internet during adolescence

**DOI:** 10.1007/s10212-023-00705-5

**Published:** 2023-05-25

**Authors:** Martina Benvenuti, Sabrina Panesi, Sara Giovagnoli, Patrizia Selleri, Elvis Mazzoni

**Affiliations:** 1 Department of Psychology, University of Bologna, P.zza A. Moro, 90, 47521 Cesena, FC Italy; 2 Istituto per le Tecnologie Didattiche (ITD) Consiglio Nazionale delle Ricerche (CRN), Via de Marini, 6, 16149 Genoa, GE Italy

**Keywords:** Adolescence, Online social support, Offline social support, Problematic Internet use, Functional Internet use, Gender differences

## Abstract

This study analyses the role of social support in Internet use, focusing on when it leads to problematic or functional use in male and female adolescents. Three research hypotheses state: (1) when offline social support is low, online social support leads to a problematic Internet use; (2) when offline social support is high, online social support leads to a functional Internet use; (3) significant differences between male and female adolescents in both the online and offline dimensions considered. Results showed that the positive social interaction factor of online social support positively predicts problematic Internet use and that the latter is negatively affected by offline social support (affectionate dimension). Furthermore, online social support predicts functional Internet use (positive social interaction factor), while offline social support has no such effect. Finally, gender differences occur: males show higher problematic Internet use, and a higher number of friends and acquaintances than females, while females show higher online and offline social support than males. Implications of this research are particularly relevant for schools (e.g., teachers), families (parents, caregivers, etc.), and policy maker, so that they can support adolescents in the construction and development of offline friendly relationships and promote a functional use of the Internet for preventing its negative effects with active educational policies.

## Introduction

The rapid spread of ICTs (Information and Communication Technologies) has rapidly pushed the Internet to become a pervasive tool used in everyday life, thanks in part to the development of new applications capable of facilitating online interactions and communications and opening the way to a wide variety of activities, particularly during adolescence (Anderson et al., 2017). The possibility of being constantly connected to the Internet strongly increases the time one spends online, and the Web can certainly be seen as a remarkably pervasive aspect of adolescents’ lives nowadays (Durak, 2018). European research has shown that adolescents use the Internet more frequently and in a more multipurpose context than children do (Livingstone et al., 2011; Casaló & Escario, 2019). The question many scholars are now attempting to answer concerns how adolescents’ online presence shapes their offline life; this could be encapsulated as follows: “Is the use of the Internet good or bad for adolescents?” Analysis of the issue has tended to follow two different research trends. On the one side, researchers have deeply analyzed the problematic aspects of the use of the Internet (Gansner et al., 2019; Throuvala et al., 2019). For instance, Valkenburg et al. (2005) showed the way in which adolescents use Social Networking Sites (SNSs), particularly as it pertains to the frequency of use, indirectly affected both psychological well-being and self-esteem. The use of SNSs depends on the frequency of positive feedback that users receive, e.g., “Likes” gained on Facebook or “Re-tweets” generated on Twitter. Results of another study showed that many individuals are starting to prefer online social support (e.g., chatting, vocal messaging) to the offline one (face-to-face interactions) since the first, more than the second, is often characterized by anonymity, fewer negative judgments, less harsh and more focused answers/reactions, and more expressive and uninterrupted communication (Walther & Boyd, 2002). Thus, the Internet is seen as a tool that could reduce adolescents’ resources (such as offline social support) rather than enhancing them. On the other hand, other scholars have tried to find confirmations of the empowerment of human abilities and skills generated by some aspects of the Web. For example, Frozzi and Mazzoni (2011) and Mazzoni and Iannone, (2014) highlighted the importance of using the Internet during life transitions to share information and/or communicate with peer groups. These relationships (both online and offline) have a great influence on the use of the Internet and its applications for males and females during adolescence and emerging adulthood. Despite the massive spread of smartphones and tablets in recent years, the use of ICTs still shows a digital divide between females and males (Van Deursen & Van Dijk, 2014). This is shown in a number of studies highlighting higher use by males (Ha & Hwang, 2014; Pujazon-Zazik et al., 2010). Cultural characteristics seem to be one of the most important factors influencing this aspect (Meelissen & Drent, 2008; Tømte & Hatlevik, 2011). For example, girls tend to use the Internet more for socializing, online shopping (Griffiths, 2015), and keeping in touch with friends (Sebre et al., 2020), while boys tend to use the Internet for longer time periods than girls, but mainly for video-gaming, to access pornographic material and keep in touch with people in general, independently from offline friendships (Griffiths, 2015).

Social relationships are fundamental during adolescence (Portt et al., 2020). Adolescents build social support through acquaintances (people they know but do not meet regularly) and friends (people they have a relationship with that goes beyond occasional encounters). These are important both in their offline and their online lives (Bastiaensens et al., 2019). Online social support, in particular during Covid-19 emergency, but also before it, thanks to the multiplicity of current social media in which it can be activated (e.g., Facebook, Instagram, WhatsApp, WeChat, YouTube), has a positive impact on adolescents’ general well-being (Luo & Hancock, 2020), but also to improve their general physical and mental health (Gilmour et al., 2020). However, this positive impact of online social support during Covid seems to be counterbalanced by the heavy Internet use during a regular lockdown day, which significantly predicts low well-being (Brandtzæg & Lüders, 2021).

Considering these aspects, this study tries to go beyond the simplistic dichotomy whereby the Internet is either good or bad. It considers the fundamental role of social support in online and offline adolescents’ relationships, and it tries to answer the question: “When do online social support and offline social support lead to problematic or functional social media use for male and female adolescents?”.

## The evolution of the concept of social support in Internet use

The concept of social support describes the assistance and protection that a person receives from others, normally by means of a social network (Rowsell et al., 2016). A relevant aspect of social support is its perception, i.e., evaluation of how real and supportive social relations are for a person (both online and offline) when they are needed (Lakey & Scoboria, 2005). During adolescence, social support, the perception of it, and social connectedness are central to peer groups and it can predict psychological well-being over time (Jose et al., 2012). Examining the broad spread of Internet communication (including SNSs) over the last few decades, it can be seen that the perception of social support, but also of the concept itself, has changed: new distinctions are needed in order to take into account the aspects of social support both in offline and in online life. Thus, based on the results of the study of Wang and Wang (2013), it could be helpful to distinguish between the two types of social support, considering that having more support in offline life as opposed to online is negatively correlated with a dysfunctional use of the Internet in general, whereas having closer relationships and greater support online as opposed to offline increases the risks of a problematic Internet use (Wang & Wang, 2013). According to the authors, the growing need to be online and to stay connected could be determined by the fact that people rely more and more on online contacts and, consequently, they predominantly construct and activate online connections. During adolescence, building social bonds with peers, online and offline, is essential to increasing social support and consequently self-esteem (Anderson et al., 2017; Argyle, 2017). Żywica and Danowski (2008) proposed the hypothesis of social enhancement versus social compensation. In their study, they found that greater extroversion and higher self-esteem are connected to self-perception of being more popular, in both online and offline life (social enhancement). By contrast, those people with lower self-esteem are perceived by other people as being less popular both in their online and in their offline lives (social compensation). Adolescents perceive and describe their increased online involvement as something normal, but also peer driven (Desjarlais & Willoughby, 2010). Various studies showed the link between Internet use and group norms (Anderson et al., 2017; Hellstrom et al., 2012, but also with aspects characteristic of adolescence such as identity exploration (Israelashvili et al., 2012), self-affirmation (Hellstrom et al., 2012), and belonging (Bryant, Sanders-Jackson & Smallwood, 2006). Studies analyzing the positive effects of significant online involvement have shown that frequent online interactions are positively associated with greater familiarity and self-disclosure (Valkenburg & Peter, 2007), and they can predict the quality of future friendships (Desjarlais & Willoughby, 2010). Within the research analyzing the relationship between Internet use and social support in adolescents’ samples, some scholars have tried to verify whether using the Internet affects their feelings and habits (Jose et al., 2012; Tzavela et al., 2015), considering both the problematic and functional side.

### *From problematic Internet use to problematic social media use*

The study of problematic Internet use suffers from the lack of a unique and shared definition of this phenomenon. Indeed, it is questionable whether the multiplicity of conceptual definitions and of tools of measurement addresses the same underlying construct (Tokunaga and Rains, 2010). Thus, trying to better clarify what problematic Internet use means is a fundamental step. The first conceptualization of Problematic Internet Use (PIU) can be attributed to Shapira et al. (2000) that described a clinically important syndrome associated with distress, functional impairment, and psychiatric disorders. This perspective was derived from studies, based on the DSM-IV definition for pathological gambling and substance dependence, associating PIU with “Internet addiction” (see Beard & Wolf, 2001; Griffiths, 1998; Young & Rogers, 1998). From this point of view, PIU is conceived as a behavioral addiction similar in character to other impulse control disorders such as gambling (Beard & Wolf, 2001; Griffiths, 1998, Young, 1998; Young & Rogers, 1998). However, an aspect that has to be taken into account is that the so-called Internet 1.0, from the late 1990s to the beginning of the 2000s, was considerably different from today: first of all, few people used computer-mediated communication; further, online life was mostly separate from the offline life; and finally, the use of the Web produced a strong spill-over effect. This meant that time spent on the computer had the potential risk to decrease traditional social relationships. The spread of SNSs characterized the advent of the Web 2.0 in which the distinction between the online and the offline world became less and less defined. Nowadays, online chatting is the most used way to talk to acquaintances and friends already characterizing the offline life networks and it can be conceived as a means of strengthening offline social ties and to socially activating further online contacts. Since the Internet seems to have become the most relevant technology pervading twenty-first century human life, research in this domain has become more and more extensive and well-articulated. In this regard, following Caplan’s (2007, 2010) theory applied to current internet interaction, this study focuses specifically on the problematic use rather than adopting a merely clinical approach such as that of addiction.

However, an important issue to also consider in the Caplan’s theory is what does “Internet use” mean? The passage from Web 1.0 to Web 2.0 determined an important change in online activities, passing from activities principally devoted to searching and finding information, to activities devoted to constructing and sharing many types of contents by means of different types of social networking sites (Briciu & Briciu, 2021; Lin et al., 2013). Using social media to share content (texts, pictures, audios, or videos); to chat with friends and acquaintances; to construct, maintain, and develop personal social capital; and to play with others has become most of online activities people do (Andreassen et al., 2016; Marciano et al., 2022; Musetti et al., 2022; Shannon et al., 2022; Vogels et al., 2022), particularly in adolescence when social relations play an important role in most of the developmental processes. Thus, considering adolescents’ use of the Internet, it could be more appropriate speaking of problematic social media use (Boer et al., 2020) in which the term “social media” involves both social networking sites and messenger platforms that represent the most used applications by adolescents along with online videogames (Wartberg et al., 2020).

Italian adolescents are not exempt from this trend in the use of the Internet. Indeed, a recent online survey conducted by Generazioni Connesse — the Italian Safer Internet Center, coordinated by the Ministry of Education and Merit — curated by Skuola.net, University of Florence and Sapienza University of Rome — CIRMPA, involving 3488 adolescents, shows that, although there is a decrease in the time spent online per day, social media is becoming increasingly the digital place where adolescents spend the most time for entertainment, the construction of networks and communities but also as primary source of information (Generazioni Connesse, 2023). Consequently, many researches have focalized their attention on the Problematic Social Media Use and its effects on many aspects of daily life, particularly during Covid-19, such as sleep-onset difficulties (Varghese et al., 2021), social media addiction, (Marengo et al., 2022), or emotional instability (Musetti et al., 2021; Marino et al., 2020).

### From functional internet use to functional social media use

Despite the fact that in recent years more and more people have become able to connect to the Internet in almost every country in the world (http://www.Internetworldstats.com/stats.htm), studies in the literature have mainly focused on the problematic/addictive side of Internet use rather than on its functional/positive use (Caplan, 2010; Davis, 2001; Kuss et al., 2013; Schønning et al., 2020). Indeed, there are still relatively few psychology-related studies which consider the Internet as a tool capable of enhancing human skills as well as acting as a functional tool for people (Mazzoni et al., 2016; Sum et al., 2009; Vella-Brodrick & Klein, 2010). Other research has focused specifically on the usage of SNSs to verify how they can lead to positive outcomes and high levels of well-being and consequently improve life satisfaction (Oh et al., 2014; Valenzuela et al., 2009). In a sample of Chinese and Taiwanese students, Liu and Tsai (2012) confirmed (as previous postulated by Desjarlais & Willoughby, 2010; Valenzuela et al., 2009) that the frequent use of SNSs can lead to higher levels of self-esteem when positive reactions are received (e.g., positive comments or many “Likes” in published posts). A positive association was also found between the number of Facebook friends and subjective well-being (Ellison et al., 2007, 2011; Kim & Lee, 2011; Steinfield, Ellison & Lampe, 2008). Ellison et al. (2007) investigated the role played by Facebook and other SNSs in a sample of college students in building and maintaining social capital, i.e., the resources associated with relationships with other people within a community. Scholars distinguish between bridging social capital and bonding social capital, with the latter referring to tighter ties involving emotional support. An intense use of SNSs was positively associated with the formation and maintenance of bridging social capital and bonding social capital, with the strongest relationships found in bridging social capital (Mazzoni and Iannone 2014). Although the presented studies are quite dated, they all focus attention on the use of social networking sites as it is the most prominent activity of adolescents in their online life. Thus, for the same reasons suggested regarding the passage from Problematic Internet Use to Problematic Social Media Use considering adolescence, as with Functional Internet Use, it should more correctly speak about Functional Social Media Use. In regard to that, some studies showed the relevance of Social Media Use during Covid-19 lockdown to stay connected with their friends and peers while practicing social distancing (Nagata et al., 2020) and to cope with feelings of loneliness and anxiety (Cauberghe et al., 2021).

## Research hypotheses

This research assumes the theoretical perspective that, in the same way as any other cultural artifact created by humans, the Internet, and particularly social media (the applications used the most by adolescents), can become either a functional or dysfunctional device during adolescence, depending on how they are used and the reasons behind their use (Mazzoni et al., 2016, 2017). Thus, based on the previously outlined theoretical background and taking into account gender differences, the online social support and the offline one are both considered as main factors that can lead to either problematic or functional social media use during adolescence. Specifically, the consequences of online social support on the recipient are expected to be positive or negative depending on the strength of their perceived support in their offline life. Social support represents the whole set of information that comes to a person through social interactions, and it transmits to the individual the feeling of being loved, esteemed, capable, and part of a network characterized by reciprocal obligations (Cobb, 1976). Since Zywica and Danowski (2008) in their “social compensation hypothesis” underlined that if online social support aims to compensate the perception of weak offline social networks, the user could develop problematic social media use, therefore:

- H1. *When offline social support is low, online social support positively predicts problematic social media use*.

By contrast, it is expected that if an individual (adolescent) has strong social bonds in his offline life, these bonds (friends and acquaintances) will be re-created in his online life (Steinfield, Ellison & Lampe, 2008), and this could predict functional social media use. Thus, it is hypothesized that adolescents who have a strong perception of their offline social support will benefit from receiving online social support.

- H2. *When offline social support is high, online social support positively predicts functional social media use*.

## Gender differences

Based on the assumptions made in previous literature, it is expected that adolescent males would have higher levels of problematic social media use and greater online social support than females. Conversely, adolescent females were expected to have higher levels of functional social media use and greater offline social support than males. Males were also expected to have a greater number of acquaintances and friends than females. Considering these aspects, this study stated the following hypothesis:

- H3. *Significant differences between male and female adolescents in both the online and offline dimensions considered*.

## Materials and method

### Data collection and procedure

The purpose of this study is to explore the impact of both online and offline social support in adolescents’ use of the Internet. The study aims to examine whether, when, and under which conditions the relation between offline and online social support leads to a problematic or a functional use of social media. For this reason, following approval by the relevant university bioethics committee, a cross-sectional study was planned based on the compilation of a self-report questionnaire performed with Qualtrics via the Web. To give more information about the research, a website has been created, so that anyone interested in participating could read the information, consult the informed consent and data processing conditions, and then proceed to fill in the questionnaire. The distribution of the questionnaire was based on the penetration of SNSs and their monthly use in Italy (https://www.statista.com/statistics/312943/social-network-penetration-in-italy/; https://www.statista.com/statistics/567335/predicted-social-network-user-penetration-rate-in-italy/). Firstly, three Italian high schools were contacted directly to participate in the research and, after receiving the consent of the School Principal, a link to the questionnaire was sent to all the students as well as to their parents so they could sign the consent form for participation and data processing.

### Sample description

After data collection, only fully completed questionnaires were selected for analysis; thus, the sample consisted of 574 adolescents (mean = 15.74, SD = 1.15), of which 303 were females (52.8%) and 271 were males (47.2%).

### Measures

The aim of this study is to investigate the relationship between constructs referring to adolescents’ offline life and a number of dimensions related to their activity online (Table 1).

**Table 1:** Dimensions for offline life and online life considered in this study

Offline life	Online life
Offline social support	Online social support
Number of friends (those with whom one has a relationship that goes beyond mere acquaintance)	Number of Friends (those with whom one has a relationship that goes beyond mere acquaintance)
Number of Acquaintances (those one knows but does not contact regularly)	Number of Acquaintances (those one knows but do not contact regularly)
	Functional Internet Use (an indirect measure of the functional social media use)
	Problematic Internet Use (an indirect measure of the problematic social media use)

As suggested by previous studies investigating online activity, the analyses were accomplished considering gender as a control variable (Anderson et al., 2017; Lakey & Scoboria, 2005; Valkenburg et al., 2006).

#### Offline and online social support

Offline and online social support were assessed by translating Wang and Wang’s offline and online social support scales (Wang & Wang, 2013) into Italian, following a forward–backward translation procedure. Each scale consists of 11 items which are the same for both offline and online social support. They address the question “If necessary, how often are you able to count on the following kinds of support?” The answers were given according to a 4-point Likert scale. Although Wang and Wang (2013) treated the scales as uni-dimensional, their instruments are based on Leung and Lee’s (2005) inventory, which distinguishes three factors: Emotional and Informational (EI), Positive Social Interaction (PSI), and Affectionate (AF), either for online and offline life. Cronbach’s alpha shows that both the scales and all their factors are reliable for the sample of adolescents (*n* = 574; see Table 2).

**Table 2:** Cronbach’s alpha of offline and online social support scales

*Dimensions*	*a*	*Items*
Offline social support	*0.92*	
Emotional and Informational	*0.90*	Someone whose advice you really want
		Someone who can give you good advice in a crisis
		Someone who can give you information to help you understand a situation
		Someone you can turn to for suggestions about how to deal with a personal problem
Positive Social Interaction	*0.90*	Someone you can get together with to relax
		Someone you can do something enjoyable with
		Someone you can do things with to help you get things off your mind
Affectionate	*0.89*	Someone who shows you love and affection
		Someone who wants you and makes you feel wanted
		Someone who comforts sincerely
		Someone you can count on to listen to you when you need to talk
Online social support	*0.96*	
Emotional and Informational	*0.94*	Someone whose advice you really want
		Someone who can give you good advice in a crisis
		Someone who can give you information to help you understand a situation
		Someone you can turn to for suggestions about how to deal with a personal problem
Positive Social Interaction	*0.93*	Someone you can get together with to relax
		Someone you can do something enjoyable with
		Someone you can do things with to help you get things off your mind
Affectionate	*0.92*	Someone who shows you love and affection
		Someone who wants you and makes you feel wanted
		Someone who comforts sincerely
		Someone you can count on to listen to you when you need to talk

*a* represents Cronbach’s alpha

#### Problematic social media use

Problematic social media use was measured using the Italian adaptation of the Generalized problematic Internet Use scale 2 (GPIU2) (Caplan, 2010), made by Fioravanti et al. (2013). It consists of 15 items answering the question: “Indicate your agreement-disagreement with the following statements.” Answers were given according to an 8-point Likert scale (1 = strongly disagree and 8 = strongly agree). Although this scale focuses attention on the Internet in general, literature (Marciano et al., 2022; Musetti et al., 2022; Shannon et al., 2022; Vogels et al., 2022) suggests that, speaking of the Internet, the use of social media has become the most of online activity people do, particularly adolescents. And as this study is focusing attention also on online social support that passes through social networking sites, the “use of the internet” can be taken as an indirect measure of the use of social media.

#### Functional social media use

The Web Useful scale (*a* = 0.90) is a short scale specifically created for this study in order to measure functional Internet use. The scale measures the persons’ perception that using the Internet helps them to carry out certain activities better. It is composed of 4 items, answering the question: “Indicate your agreement-disagreement with the following statements”: (1) “being connected increases my ability to reach certain goals,” (2) “being connected improves my productivity,” (3) “being connected is useful for carrying out my activities,” (4) “being connected improves my performance,” and is based on a 7-point Likert scale from 1 = strongly disagree to 7 = strongly agree. For the same reasons that, particularly considering adolescents, problematic Internet use is an indirect measure of problematic social media use, functional Internet use is an indirect measure of functional social media use.

#### Number of online contacts and acquaintances

Participants were also asked to answer specific questions concerning friends and acquaintances. As regards friends, the question was: “if you had to quantify the number of friends you have (people with whom you have a relationship that goes beyond mere acquaintance), how many would you say you have?” A similar question was also asked regarding the number of acquaintances: “if you had to quantify your acquaintances (people you know but do not contact regularly), how many would you say acquaintances you have?”.

### Data analysis

After calculating the reliability of the scales of PIU (0.92) and FIU (0.90), Table 3 shows Spearman’s rank-order correlation carried out to evaluate the relationships between the considered dimensions.

**Table 3:** Correlations of all the considered dimensions

Measure	Mean	SD	1	2	3	4	5	6	7	8	9	10
1. Offline EI	3.71	0.96	1									
2. Offline PSI	4.00	0.89	0.53**	1								
3. Offline AF	3.87	1.00	0.67**	0.62**	1							
4. Online EI	3.32	1.10	0.36**	0.29**	0.32**	1						
5. Online PSI	3.57	1.07	0.22**	0.31**	0.22**	0.71**	1					
6. Online AF	3.28	1.14	0.35**	0.34**	0.45**	0.79**	0.72**	1				
7. FIU	14.2	6.18	0.02	0.01	0.03	0.22**	0.27**	0.21**	1			
8. PIU	3.19	1.43	− 0.12**	− 0.11*	− 0.16**	0.19**	0.22**	0.16**	0.25**	1		
9. No. of Online Friends	3.03	1.40	0.11**	0.19*	0.14**	0.14**	0.11**	0.15**	0.21**	0.02	1	
10. No. of Online Acquaintances	4.14	1.38	0.05	0.16**	0.14**	0.10*	0.05	0.10*	0.15**	0.05	0.42**	1

*p < 0.05, **p < 0.01

The analysis shows that all factors of offline and online social support are correlated with each other. Furthermore, while factors representing online social support are positively related to PIU, those characterizing offline social support are negatively correlated with the same factor. FIU, however, has no correlation with Offline social support factors, though it is positively related to those of online social support. In addition, the total number of online friends is positively correlated with all factors of online and offline social support, and with FIU. Lastly, the number of acquaintances is positively correlated with offline social support PSI and AF, online social support EI and AF, and FIU. However, the number of friends has no correlation with offline social support EI, online social support PSI, or PIU.

#### Hypotheses testing

The first hypothesis H1 (*when offline social support is low, online social support positively predicts PIU*) was tested using a Multiple Regression Analysis conducted by means of a General Linear Model (GLM) procedure with PIU as a dependent variable, gender as a factor, and offline/online Indexes, number of friends, and number of acquaintances as predictors (Table 4).

**Table 4:** Multiple Regression Analysis conducted by means of GLM procedure, dependent variable: problematic Internet use

Gender (*F* = *3.83)*			
	*b*	*SE*	*t*
Offline EI	− 0.078	0.084	− 0.935
Offline PSI	− 0.120	0.087	− 1.37
Offline AF	− 0.258	0.091	− 2.82**
Online EI	0.161	0.093	1.74
Online PSI	0.173	0.085	2.03*
Online AF	0.128	0.095	1.35
No. of Online Friends	− 0.034	0.048	− 0.72
No. of Online Acquaintances	0.064	0.046	1.385

*p < 0.05, **p < 0.01

The results show that H1 is partially confirmed: online social support, in its positive social interaction factor, positively predicts PIU, while offline social support, in its affectionate factor, negatively affects it.

H2 (*when offline social support is high, online social support positively predicts FIU*) was tested using a Multiple Regression Analysis conducted by means of a General Linear Model (GLM) procedure with FIU as dependent variable, gender as a factor, and offline/online Indexes, number of friends, and number of acquaintances as predictors (Table 5).

**Table 5:** Multiple regression analysis conducted by means of GLM procedure, dependent variable: functional Internet use

Gender (*F* = 8.87**)			
	*b*	SE	*t*
Offline EI	− 0.096	0.359	− 0.266
Offline PSI	− 0.600	0.374	− 1.604
Offline AF	0.222	0.392	0.565
Online EI	0.446	0.397	1.122
Online PSI	1.340	0.366	3.660**
Online AF	− 0.122	0.406	− 0.300
No. of Online Friends	0.563	0.204	2.753**
No. of Online Acquaintances	0.373	0.199	1.880

*p < 0.05, **p < 0.01

Results also partially confirm the second hypothesis: even though online social support predicts FIU specifically in its positive social interaction factor, offline social support does not affect it. Furthermore, the total number of friends is a good predictor of FIU. A significant difference between genders was also found.

#### Gender differences

Gender differences were tested using a multivariate analysis of variance (Table 6).

**Table 6:** MANOVA test of measured dimensions between females and males

Measures	*F*	Male (Mean ± SD)	Female (Mean ± SD)
Offline EI	25.28**	3.49 ± 0.06	3.90 ± 0.06
Offline PSI	9.17**	3.87 ± 0.05	4.10 ± 0.05
Offline AF	26.84**	3.63 ± 0.06	4.06 ± 0.06
Online EI	6.03**	3.20 ± 0.07	3.43 ± 0.06
Online PSI	0.025	3.56 ± 0.07	3.58 ± 0.06
Online AF	4.74**	3.17 ± 0.07	3.38 ± 0.07
Functional Internet Use	17.65**	15.4 ± 0.38	13.2 ± 0.36
Problematic Internet Use	8.10**	3.37 ± 0.09	3.03 ± 0.08
No. of Online Friends	49.39**	3.47 ± 0.08	2.66 ± 0.08
No. of Online Acquaintances	8.93**	4.34 ± 0.08	3.99 ± 0.08

*p < 0.05, **p < 0.01

Adolescent males have significantly higher levels of PIU and FIU than adolescent females, as well as a greater total number of friends and acquaintances. However, adolescent females have the highest scores in all factors of both offline and online social support, with the exception of online PSI, though this is not significant.

## Discussion

This study seeks to answer the question: “When do online social support and offline social support lead to problematic or functional social media use for female and male adolescents?” Following the hypotheses made, a variety of aspects were verified in a wide sample of adolescent Italian males and females. The correlation analysis, the Multiple Regression Analysis conducted by means of a General Linear Model (GLM) procedure, and the MANOVA allowed us to gain a deeper understanding of the dynamics analyzed. Concerning the first hypothesis (H1), which states that online social support positively predicts PIU only when offline social support is low, results show that the affective factor of offline social support (the factor most related to “feeling loved”) negatively affects PIU, and that the positive social interaction of online social support (i.e., the factor related to “not feeling alone”) positively predicts PIU. Considering the compensation theory (Mazzoni et al., 2016; Zywica & Danowski, 2008), feeling loved in their offline lives protects adolescents from the risk of problematic social media use, which is likely to be linked to the search for online contacts in order to avoid feeling alone. However, the search for positive online social interaction could reflect the need to compensate for the lack of satisfaction (or satisfactory relationships) in one’s offline life, although substituting actual affectionate relationships with a larger number of relationships that are more ephemeral in nature could lead to an increase in problematic online behavior and social media use. Affectionate scores for both PIU and offline social support show significant gender differences: adolescent males have higher levels of PIU compared to females, while the latter have higher scores in the affectionate factor of offline social support. This could mean that since adolescent males tend to have fewer offline social bonds that provide affection than females do, they tend to spend much more time on the Web, particularly on social media, not necessarily to search for the same type of relationships in the online context — as seen in Griffiths (2015) — but simply to accrue more ephemeral interactions and thus avoid feeling alone. Nevertheless, the results reveal gender differences in all dimensions (both online and offline), except for positive social interaction factor in online social support. Male and female adolescents seem to have the same perception of using the Internet, and thus social media, positively, which is not related to the social relationships they have in their offline lives. However, it would appear they do not compensate for shortcomings in the number of offline social ties, but simply search for online enjoyment. This enjoyment, particularly in the case of adolescent males, derives from the number of friends which make up their online social ties.

Turning to H2 (online social support positively predicts functional social media use only when offline social support is high), the positive social interaction factor of online social support (i.e., the factor related to enjoyment) predicts FIU, while offline social support does not affect FIU. So, it could be said that adolescents with positive online social relationships who feel relaxed are able to enjoy doing things together and find help when it is needed, perceiving their use of social media as functional. The results obtained, particularly those concerning the influence of positive social interaction on FIU, resonate with the findings of Walther and Boyd (2002), i.e., online social support is characterized by less harsh and more focused responses, fewer negative judgments, anonymity, and more expressive and uninterrupted communication. This result can also be related to the results of Brandtzaed and Lüders (2021), and from this point of view, we could say that online social support has a positive impact on well-being but only when the use of the Internet is perceived as functional (in the sense of not heavy Internet use). Moreover, this result confirms the finding of Liu and Tsai (2012), in which the frequent use of SNSs leads to higher levels of self-esteem when positive reactions were received. The results concerning the influence of the number of online friends on FIU are linked to those of Rowsell et al. (2016), but only regarding close ties. Indeed, their findings showed that close ties and non-close ties provide equal perceptions of social support. This study, however, found that only online friends (where ties are regarded as close), and not online acquaintances, affect functional social media use.

Finally, as regards the gender differences found in H2 but not in H1, by considering the multivariate analysis, the results of this study can be linked with those of other studies (Laconi et al., 2018). Here, females use the Internet largely to socialize and chat in order to maintain the relationships established in their offline lives. Indeed, in all the dimensions of online and offline social support, except for online PSI, females have higher scores than males. How then can be explained a similar relationship between online positive social interaction with, on the one hand, PIU and, on the other, FIU? It is likely that problematic and functional Internet use, and thus problematic and functional social media use, are not the two poles of a continuum going from negative to positive use of social media. In the context of this study, social media could simply be perceived as functional since they allow adolescents to avoid feeling alone. At the same time, however, this could, in the case of low offline social support, particularly in the affective dimension, lead to problematic interaction with the social media.

## Conclusion

One of the aims of this study was to redress the imbalance in the literature whereby dysfunctional use of social media is emphasized excessively compared to their functional use; this was done by providing an understanding of the relationships which exist between online and offline life. More specifically, the main objective was to focus on the roles played by offline and online social support in determining problematic social media use during adolescence, but also examining the factors that lead adolescents, both males and females, to functional social media use so as to achieve the goals in daily life. The findings highlight the way in which strong affective relationships in an individual’s offline life are instrumental in preventing problematic use of social media, particularly for males. Furthermore, the results show that creating positive online relationships could increase the risk of developing problematic social media use, especially when offline social interactions are perceived as infrequent or of poor quality. At the same time, the results also highlight the relevance of online social support, particularly positive social interaction, for adolescents’ perception of functional social media use. From this point of view, problematic and functional use of social media could be seen as coexistent and integrated dimensions rather than separate poles of a continuum going from negative to positive.

Having a large amount of positive social interaction in online life promotes the perception, in both male and female adolescents, of using social media in a functional way, particularly where they interact with friends. These results could have certain practical implications. The opportunity provided by technological devices like smartphones and tablets for always-on access to the Internet practically anywhere has led to an increasing amount of apprehension about the possible effects of such massive usage. However, the findings of this study highlight that social media cannot be thought of simply as addictive or risky per se; their effects are actually strongly related to adolescents’ offline life, particularly to their offline social connections. Interventions aiming to reduce dysfunctional outcomes of social media use should work on enhancing factors related to offline social support during adolescence rather than focusing simply on device use or time spent accessing the Web.

## Practical Implications, Limitations, and Future Directions

A first practical implication of this study is the understanding of the relevance of offline social support and of online social support to enhance a functional social media use and prevent a problematic use in adolescents. Results show that in order to prevent the problematic use it is important to promote and support offline relational networks in such a way as to sustain the construction of strong (friends) and positive online relational networks. At the same time, this study underlines the promotion of educational paths in which the difference between offline and online social support is clear. The latter in particular is characterized by the instability of latent ties and not-close ties (Haythornthwaite, 2005), and a number of dysfunctional effects such as Phubbing (Błachnio & Przepiorka, 2019), Ghosting (Rad & Rad, 2018), or Vamping (Vernon et al., 2018); it could therefore be considered important to develop a critical sense able to contrast these effects and develop stable and secure friendly relationships. This last implication is particularly relevant for schools (teachers) and families (parents, caregiver), so that they can support adolescents in the construction and development of offline friendly relationships and promote a functional use of social media, thus preventing its negative effects. Moreover, these results could offer useful insights for policy makers, to be able to build educational policies that prevent the dysfunctional use of social media and promote informed use.

A limitation of this study is the use of the Generalized problematic Internet Use scale 2 (GPIU2) and of the Functional Internet Use Scale as indirect measures of respectively problematic and functional social media use. Despite the fact that, as we have introduced, literature shows that today use of the Internet, particularly during adolescence, is mostly related to social media use, future research with direct measures of social media use will allow a deeper understanding of the results obtained by this study.

Probably a longitudinal study would have provided more complete data for understanding the role played by online and offline social support in the problematic or functional social media use of male and female adolescents. However, the rapid changes characterizing the Web and its applications (e.g., Instagram, Facebook, Twitter, LinkedIn), but also the devices used to connect to the Internet (e.g., Smartphones, Tablets), would not have allowed continuity and availability of data collection in a potential longitudinal study. Indeed, if a longitudinal study were hypothesized (e.g., over a period of 5 years) with a group of adolescents, their habits regarding the use of social media and its applications may suddenly change in the meantime and there would consequently be difficulties in considering and assessing the same factors all along the period of the study.

## Data Availability

Data are available under request to authors.

## Declarations

This study was performed in line with the principles of the Declaration of Helsinki. Approval was granted by the ALMA MATER STUDIORUM – University of Bologna bioethics committee (https://www.unibo.it/en/research/research-facilities/ethics-committees/bioethics-committee).
